# Matrix Metalloproteinases: Pathophysiologic Implications and Potential Therapeutic Targets in Cardiovascular Disease

**DOI:** 10.3390/biom15040598

**Published:** 2025-04-17

**Authors:** Daniela Maria Tanase, Emilia Valasciuc, Ioana-Bianca Anton, Evelina Maria Gosav, Nicoleta Dima, Andrei Ionut Cucu, Claudia Florida Costea, Diana Elena Floria, Loredana Liliana Hurjui, Claudia Cristina Tarniceriu, Manuela Ciocoiu, Mariana Floria

**Affiliations:** 1 Department of Internal Medicine, “Grigore T. Popa” University of Medicine and Pharmacy, 700115 Iasi, Romania; daniela.tanase@umfiasi.ro (D.M.T.); anton_bianca14@yahoo.com (I.-B.A.); dr.evelinagosav@gmail.com (E.M.G.); nicoleta.dima@umfiasi.ro (N.D.); diana-elena.iov@d.umfiasi.ro (D.E.F.); floria.mariana@umfiasi.ro (M.F.); 2 Internal Medicine Clinic, “St. Spiridon” County Clinical Emergency Hospital, 700111 Iasi, Romania; 3 Department of Biomedical Sciences, Faculty of Medicine and Biological Sciences, “Stefan cel Mare” University, 720229 Suceava, Romania; andrei.cucu@usm.ro; 4 Department of Neurosurgery, “Prof. Dr. Nicolae Oblu” Emergency Clinical Hospital, 700309 Iasi, Romania; 5 Department of Ophthalmology, “Grigore T. Popa” University of Medicine and Pharmacy, 700115 Iasi, Romania; costea10@yahoo.com; 6 2nd Ophthalmology Clinic, “Prof. Dr. Nicolae Oblu” Emergency Clinical Hospital, 700309 Iasi, Romania; 7 Institute of Gastroenterology and Hepatology, “St. Spiridon” County Clinical Emergency Hospital, 700111 Iasi, Romania; 8 Department of Morpho-Functional Sciences II, Physiology Discipline, “Grigore T. Popa” University of Medicine and Pharmacy, 700115 Iasi, Romania; loredana.hurjui@umfiasi.ro; 9 Hematology Laboratory, “St. Spiridon” County Clinical Emergency Hospital, 700111 Iasi, Romania; 10 Department of Morpho-Functional Sciences I, Discipline of Anatomy, “Grigore T. Popa” University of Medicine and Pharmacy, 700115 Iasi, Romania; cristinaghib@yahoo.com; 11 Hematology Clinic, “Sf. Spiridon” County Clinical Emergency Hospital, 700111 Iasi, Romania; 12 Department of Pathophysiology, Faculty of Medicine, “Grigore T. Popa” University of Medicine and Pharmacy, 700115 Iasi, Romania; manuela.ciocoiu@umfiasi.ro

**Keywords:** matrix metalloproteinases, MMPs, cardiovascular disease, CVDs, atherosclerosis, heart failure

## Abstract

Matrix metalloproteinases (MMPs) are a family of zinc-dependent endopeptidases that play a crucial role in extracellular matrix (ECM) remodeling and are implicated in the pathogenesis of various cardiovascular diseases (CVDs). Their dysregulation has been linked to atherosclerosis, myocardial infarction (MI), heart failure (HF), and aortic stenosis, contributing to vascular inflammation, plaque destabilization, and adverse cardiac remodeling. Recent research highlights MMPs’ involvement beyond ECM degradation, influencing lipoprotein metabolism, inflammatory signaling, and intracellular processes critical for cardiovascular homeostasis. Despite their pathological role, MMPs remain promising therapeutic targets, with pharmacological inhibitors, gene therapy, and tissue inhibitors of metalloproteinases (TIMPs) emerging as potential interventions. However, the clinical translation of MMP-targeting therapies remains challenging due to off-target effects and complex regulatory mechanisms. This review provides an updated synthesis of the molecular mechanisms, disease-specific roles, and therapeutic implications of MMPs in cardiovascular pathology, aiming to bridge the gap between fundamental research and clinical applications.

## 1. Introduction

Matrix metalloproteinases (MMPs) comprise a family of over 20 zinc-dependent endopeptidases with various functions, most notably in the degradation of structural proteins within the extracellular matrix (ECM). Even minor alterations in MMPs’ activity can initiate the mechanisms involved in the pathogenesis of cardiovascular diseases (CVDs) [1,2]. Emerging research has highlighted the role of MMPs in a broad spectrum of cardiovascular conditions, including atherosclerosis (AS), hypertension, cardiac hypertrophy, heart failure (HF), aortic valve stenosis (AVS), aneurysm formation, and increased cardiovascular mortality among individuals presenting with coronary artery disease (CAD) [3,4,5]. Furthermore, the dysregulation of MMPs has been linked to numerous non-cardiovascular diseases, including cancer and arthritic disorders [2], emphasizing their broader pathophysiological significance.

Their role in chronic CVDs extends beyond ECM remodeling, as they also influence lipid metabolism by modulating lipoproteins and their receptors, key components of inflammatory and atherogenic pathways [4,6]. Notably, a deficiency in MMP-2 has been linked to impaired cardiac metabolism and altered ECM remodeling, including reduced collagen turnover, which may contribute to matrix dysfunction and risk of atheromatous plaque rupture [4,6].

The interplay between MMPs and CVD reflects a deeper connection at the level of myocyte metabolism, intracellular signaling, and disease pathophysiology. Understanding these multifaceted roles in the key functions of MMPs in cardiovascular health offers significant potential for identifying novel therapeutic targets aimed at mitigating metabolic dysregulation and improving outcomes in cardiovascular disorders [4].

This review aims to explore the expanding roles of MMPs in CVDs, emphasizing recent research into their influence on metabolic regulation, myocytes signaling, and pathophysiologic implications within various cardiac diseases. By unraveling these complex interactions, we can advance our understanding of CVD mechanisms and uncover new opportunities for personalized treatment strategies targeting MMPs metabolism and related pathways specific to each distinct cardiac tissue.

## 2. Structure, Activation, and Function of MMPs

MMPs belong to a superfamily consisting of 28 endopeptidases, including 23 human members. Together with members of the Disintegrin and Metalloproteinase (ADAM) and Disintegrin and Metalloproteinase with Thrombospondin motifs (ADAMTS), MMPs form a broader group of metallopeptidases [7]. Based on their substrates, MMPs are categorized into several families, including collagenases, membrane-type MMPs, gelatinases, metalloelastase, matrilysins, stromelysins, enamelysins, and an unclassified subgroup [1,8] as follows: collagenases (MMP-1, MMP-8, MMP-13, MMP-18), gelatinases (MMP-2 and MMP-9, 10, 11), stromelysins (MMP-3, MMP-10, MMP-11, MMP-19, and MMP-27), matrilysins (MMP-7 and MMP-26), MT-MMPs (MMP-14 MMP-15 MMP-16 MMP-4/MMP-17 MMP-5/MMP-24 MMP-6/MMP-25), and ungrouped MMPs (MMP-12 MMP-20 MMP-21 MMP-22 MMP-23 MMP-28) [7,9,10] (Table 1).

MMPs are classified into two main categories based on their molecular functions and positions: secreted and membrane-anchored MMPs, also known as membrane-type (MT) MMPs [8,9]. The latter can be attached to cell membranes through different mechanisms, including a COOH-terminal transmembrane domain (MMP-14, MMP-15, MMP-16, and MMP-24), a glycosylphosphatidylinositol (GPI) anchor (MMP-17 and MMP-25), or by an N-terminal signal anchor (MMP-23A and B) [4,9].

### 2.1. Structure

**Figure 1:**
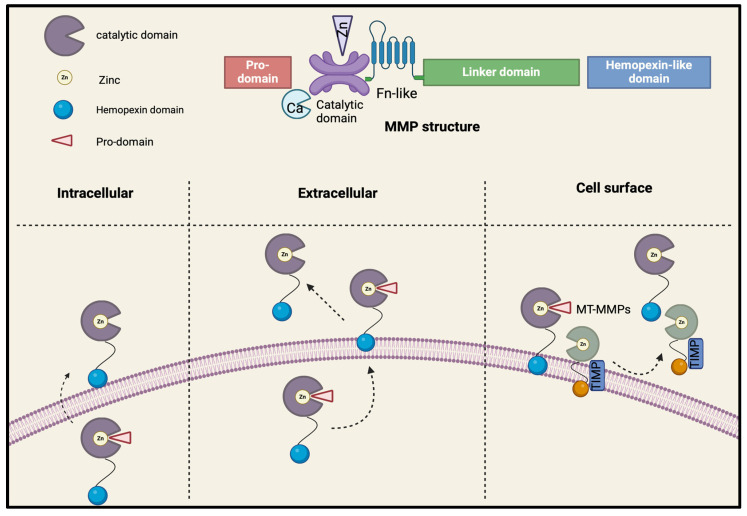
MMP structure and types of activation. Membrane-type matrix metalloproteinases (MT-MMPs); tissue inhibitors of MMPs (TIMPs); fibronectin (FN).

Structurally, MMPs are composed of three primary conserved domains that contribute to their structural and substrate specificities [1]. The catalytic domain contains two zinc ions and at least one calcium ion, which are essential for MMP stability and function [9]. The hemopexin-like (HPX) C-terminal domain, connected to the catalytic region by a flexible hinge with three histidine residues, is named after its sequence similarity to hemopexin, a plasma protein involved in heme binding and transport [9]. While the catalytic domain is highly conserved, the HPX domain exhibits significant variation, making it a distinctive feature of each metalloprotease family member. The pro-peptide region contains a cysteine residue (part of the cysteine switch, with a thiol group) that interacts with the catalytic zinc in the active locus, preventing substrate binding and keeping MMPs in their inactive zymogen forms [1,4,7,9]. MMPs are initially produced in an inactive form and are activated upon the cleavage of the pro-peptide domain [1] (Figure 1). This characteristic classifies MMPs within the zinc peptidase family, also known as the metzicin family [1,2,11,12].

### 2.2. Activation and Inhibition

The activation of MMPs is a multifaceted process, with various mechanisms identified over time by researchers. One such mechanism is known as “cysteine switch”, in which MMPs are synthesized and secreted as inactive proenzymes, also known as zymogens [1,4,7,13,14,15]. In this form, the catalytic site of the enzyme is blocked by an autoinhibitory hydrophobic pro-peptide domain that contains a cysteine residue that binds to the catalytic zinc ion and prevents the enzyme from becoming active [7]. Specifically, the pro-domain contains Cys92, a switch loop, revealing a distinct consensus sequence (PRCGVPDV) that is highly conserved [1].

A second process of MMPs’ activation is proteolytic cleavage. In this process, MMPs are activated when the pro-peptide domain is cleaved by proteolytic enzymes, such as other MMPs, plasmin, furin-like, or serine proteases [1,4,13,15]. Once the pro-peptide is removed, the catalytic zinc site becomes exposed, enabling MMP to engage in substrate degradation [15]. Notable examples of this activation are demonstrated by the furin activation site of MMP-11, MMP-21, and MMP-28 [4,15].

Another key method of activation occurs at the cell surface, where MT-MMPs, anchored to the cell membrane, can directly activate other MMPs by cleaving them into their active forms [15]. This localized activation is crucial for tissue remodeling within specific microenvironments [15], with MMP-14 serving as an example by activating pro-MMP-2 and pro-MMP-13 [15]. Environmental triggers also contribute to MMP activation. Various factors, such as inflammatory cytokines, growth factors, oxidative stress, or mechanical stress, can upregulate MMPs’ expression and promote their activation during processes like wound healing, inflammation, and tissue repair [1,4,9,10]. Moreover, the existence of nonproteolytic agents, such as detergents, oxidants, bioactive peptides, hemodynamic forces, cytokines, and reactive oxygen–nitrogen species (RONS), can also contribute to MMPs’ activation by disrupting the bond between cysteine thiol and zinc [13].

Likewise, hypochlorous acid and peroxynitrite can also produce the activation of several MMPs, including MMP-1, MMP-2, MMP-7, MMP-8, and MMP-9 [4]. MMPs have a key catalytic role that is regulated by tissue inhibitors of MMPs (TIMPs), a group of endogenous proteins that modulate MMPs’ activity. Research in the 1970s first revealed the inhibitory role of TIMPs, when a specific collagenase enzyme was discovered in human skin and animal cartilage [9]. This finding was important as it marked the identification of TIMP-1, the first TIMPs family member whose activity was studied regarding aortic localization [7,9]. Shortly thereafter, three additional homologous, TIMP2, -3, and -4, were identified [16]. TIMPs primarily interact with the HPX domain of MMPs through the C-terminal region at the majority of metalloproteases, except in the case of MMP-6, MMP-7, and MMP26 [17], while their N-terminal region targets the zinc ion in the MMP catalytic domains. This interaction leads to the formation of various inhibitory complexes consisting of a stoichiometric ratio of molecules [17].

The inhibition of MMP activity is extensively studied from various perspectives, as recent clinical research has revealed challenges in inhibiting the catalytic domain [17]. Exosites are secondary binding sites on enzymes that are distinct from the catalytic site and play a role in substrate recognition or enzyme regulation [17]. Ectosites refer to regions on the enzyme surface that are accessible from the extracellular environment and can also mediate interactions with substrates or inhibitors [17]. As a result, alternative inhibition sites, such as exosites and ectosites, are explored as additional targets [17]. One promising target is the HPX domain, which demonstrates greater specificity for amino acid sequences adjacent to the catalytic domain [17]. In addition to their role in MMP inhibition, TIMPs serve other functions, such as attaching to cell surface receptors [7]. Except TIMP-1, which is secreted by most human cells, the other three TIMPs have a more tissue-specific expression [7]. TIMP-1 inhibits a broad range of MMPs, including MMP-1, MMP-9, and pro-MMP-9, but it does not inhibit MMP-14, MMP-16, MMP-18, MMP-19, MT1-MMP, MT2-MMP, MT3-MMP, and MT5-MMP [7]. TIMP-2 selectively inhibits MMP-2, and to a smaller extent MMP-3 and MMP-7 [7], while TIMP-3 binds to the extracellular matrix and inhibits membrane-secreted MMPs such as the ADAM and ADAMTS families [7]. TIMP-3 attaches to the extracellular matrix and inhibits membrane-bound MMPs, especially MMP-2 and MMP-9 [7]. TIMP- 4 functions similarly to TIMP-1 but also inhibits MMP-2 and MMP-26 and is involved in cardiovascular tissue regulation [7].

Regarding regulation by oxidation and post-translational modifications, such as phosphorylation, the modulation of MMP activity can take place, either by enhancing or inhibiting their function. For instance, reactive oxygen species (ROS) may activate MMPs by disrupting the cysteine switch mechanism, leading to increased MMP-9 activity, tissue damage, and impaired wound healing [7,13].

Maintaining a balance between MMP activation and inhibition is essential for normal physiological processes, such as wound healing, tissue remodeling, and embryogenesis [7]. Excessive MMP activation is associated with diseases such as cancer, cardiovascular diseases (e.g., hypertension, atherosclerotic plaque formation and instability, aortic aneurysms), arthritis, varicose vein wall remodeling, and chronic inflammation, all characterized by uncontrolled matrix degradation [1]. Conversely, excessive MMP inhibition can result in impaired tissue remodeling and healing, contributing to conditions like fibrosis [13].

### 2.3. MMPs Tissue Expression

MMPs are expressed in a wide range of tissues, where they facilitate precise structural remodeling of the ECM. These zinc-dependent endopeptidases are essential for tissue development during various physiological stages, such as the embryogenetic process, angiogenesis, and wound healing, contributing to the development of structures like the heart, lungs, bones, cartilages, and membranes [7]. They are particularly important during pregnancy, where precise tissue remodeling is required [7]. Under normal conditions, MMPs’ expression in tissues remains low and tightly regulated. However, this expression is significantly upregulated in response to inflammation, tissue injury, or the presence of specific growth factors, which initiate the transcription of MMP genes [18]. MMPs such as 1, 2, 3, 7, 8, 9, 13, and 14 become overexpressed across multiple sites in pathological states, indicating their involvement in various disorders [7].

Regarding cardiovascular pathology, MMP-2 and MMP-9, also known as gelatinase A and B, act as key contributors to the development of atherosclerosis, myocardial infarction (MI), HF, and coronary thrombosis [18]. MMP-2, in particular, is widely distributed within cardiomyocytes and in various subcellular compartments, including the sarcomere, mitochondria, mitochondria-associated membranes, sarcoplasmic reticulum, and caveolae, with substantial accumulation in the nuclei [18]. This zinc- and calcium-dependent protease participates in cell migration and inflammation, being able to break down components of the contractile apparatus, including sarcomeric proteins, such as troponin I and myosin light chain-1 [18]. In states of oxidative stress, induced by ischemia–reperfusion (IR) injury or the excessive production of ROS, MMP-2 becomes pathologically activated [19,20]. Peroxynitrite (ONOO^−^), a potent oxidant derived from the ischemic myocardial tissue, is one of the most significant ROS in the cardiovascular system and has the ability to straightforwardly activate MMP-2 [19].

Within the cardiomyocyte endoplasmic reticulum, MMP-2 enhances the degradation of junctophilin-2 (JPH-2), a structural membrane-binding protein capable of anchoring T-tubules to the sarcoplasmic reticulum, thus facilitating synchronized calcium-induced calcium release required for the key process of the myocardial excitation–contraction mechanism [19]. The MMP-2-mediated cleavage of JPH-2 during myocardial IR injury contributes to an impaired cardiac muscle contraction [21]. Additionally, cytosolic MMP-2 cleaves glycogen synthase kinase (GSK)-3β, a ubiquitously expressed serine/threonine kinase involved in regulating various metabolic processes, thereby enhancing its enzymatic activity during oxidative stress [21]. In platelets, MMP-2 also exerts functional effects by cleaving talin, a cytoskeletal protein that regulates glycoprotein IIb/IIIa activation [4]. This promotes platelet activation and contributes to thrombotic processes [4].

The activity of MMP-2 is tightly regulated by TIMPs. TIMP-2 is broadly expressed across various tissues without specificity and forms part of a critical regulatory axis with MMP-14 (also known as MT1-MMP), a membrane-anchored metalloproteinase present in coronary arteries and left ventricular tissue. MMP-14 plays a key role in the extracellular activation of pro-MMP-2 by forming a ternary complex with TIMP-2 and zymogen MMP-2 at the cell membrane [3,4,22,23,24]. However, this activation can be suppressed by TIMP-4, a cardiac-specific TIMP localized to thin myofilaments of cardiomyocytes. TIMP-4 binds to MMP-14 and inhibits its enzymatic activity, forming a separate ternary complex (MMP-14-TIMP-4-pro-MMP-2) that prevents MMP-2 activation [25,26]. This dynamic interaction between MMP-14, TIMP-2, and TIMP-4 highlights the delicate balance required for ECM regulation in the heart.

MMP-14 itself is also subject to intracellular modulation. It can be internalized through caveolae- or clathrin-coated pits and undergoes regulation via phosphorylation at tyrosine (Tyr) 573 residue embedded in its cytoplasmic tail [4]. Impaired Tyr phosphorylation may lead to a reduction in MMP-14 activity, which has been linked to diminished cell division [4]. Recent research has demonstrated that human chondrocytes not only produce and secrete MMP-13 but also rapidly internalize it through the low-density lipoprotein receptor-related protein 1 (LRP1) [27]. This endocytic process involves the binding of both proMMP-13 and active MMP-13 to specific clusters on LRP1 via the HPX domain of MMP-13 [27]. Interestingly, MMP-13 shares these binding sites with other molecules such as ADAMTS-4, ADAMTS-5, and TIMP-3, leading to their co-endocytosis, thereby contributing to the maintenance of ECM homeostasis [27].

Other TIMPs exhibit tissue-specific distribution and functional significance. TIMP-3 is bound to the ECM and is notably present in the basal membranes of the kidneys and eyes, while TIMP-2, as noted, is expressed ubiquitously without tissue specificity [7].

Another membrane-anchored MMP, MMP-15 (MT2-MMP), is expressed in fibroblasts and leukocytes targeting substrates such as laminin, fibronectin, entactin, perlecan, and aggrecan, and it serves as a confirmed activator for pro-MMP-2 [28]. Due to its position at the cell surface, MMP-15 is a pivotal factor in site-specific proteolysis, although its precise role in cardiovascular pathology remains poorly defined [29].

Altogether, the coordinated expression and regulation of MMPs and TIMPs are vital for maintaining cardiac structure and function.

## 3. MMPs’ Interplay in Cardiovascular Diseases

MMPs play a central role in the structural and functional remodeling of cardiovascular tissues. Their ability to degrade ECM components makes them key regulators of vascular integrity, myocardial structure, and valvular function [3,4]. However, an imbalance between MMP activity and its natural inhibitors, TIMPs, contributes to the progression of several cardiovascular diseases, including atherosclerosis, MI, HF, and aortic stenosis [30] (Table 2). A few MMP knockout mouse models were developed over time in order to elucidate the specific roles of individual MMPs in cardiovascular diseases. Among most studied is the MMP-2 knockout (MMP-2^−^/^−^) mouse, which has demonstrated impaired cardiac remodeling and mithocondrial dysfunction [31,32]. Similarly, MMP-9 knockout (MMP-9^−^/^−^) mice exhibit reduced atherosclerotic lesion formation and improved plaque stability, emphasizing its proinflammatory and matrix-degrading functions in atherogenesis [33]. In addition, MMP-12^−^/^−^ mice exhibit a reduction in elastin degradation and monocyte recruitment within atherosclerotic lesions, indicating a significant role in ECM and arterial wall remodeling [34,35]. By mediating inflammatory responses, tissue degradation, and fibrosis, MMPs influence disease onset, progression, and complications. Understanding the mechanistic involvement of MMPs in these conditions is crucial for developing targeted therapeutic interventions to mitigate cardiovascular damage and improve clinical outcomes.

### 3.1. MMPs in Atherosclerosis and Coronary Artery Disease

Atherosclerosis is a chronic inflammatory disease characterized by a gradual buildup of lipids and inflammatory cells within the intima of large arterial walls [7,12]. It is recognized as the leading cause of MI, ischemic stroke, and peripheral artery disease, making it a major contributor to global morbidity and mortality [7,44]. Atherosclerosis is also the primary cause of CAD, which involves the narrowing or blockage of coronary arteries due to plaque formation, thus leading to endothelial dysfunction [7,44].

Over the past few years, MMPs have been extensively studied in human atherosclerotic plaques. These endopeptidases contribute to the development and destabilization of atherosclerotic plaques by breaking down key structural components like fibrillar collagen and matrix proteins, which are essential for maintaining the strength and integrity of the vessel wall [45]. By degrading the ECM, particularly in vulnerable regions, MMPs increase the risk of plaque rupture, which can trigger life-threatening events like acute coronary syndrome [46]. Vulnerable plaques are defined by features such as a thin fibrous cap, large lipid core, inflammatory cell infiltration, intraplaque hemorrhage, remodeling, and neovascularization, all of which heighten the risk of rapid plaque progression and thrombotic complications [47].

**Figure 2:**
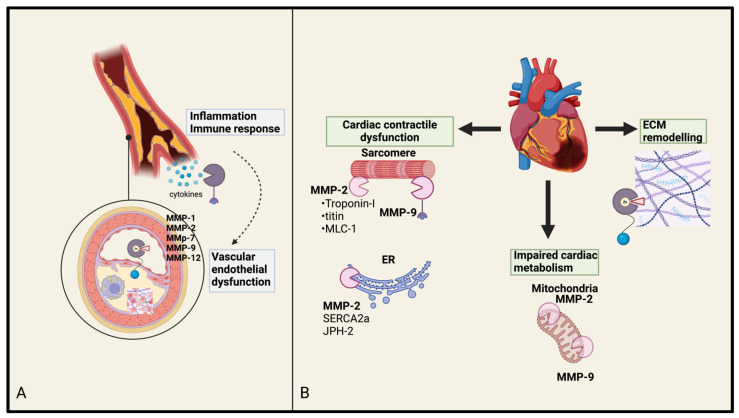
(**A**) MMP in atherosclerosis: MMPs contribute to plaque formation and rupture by degrading the extracellular matrix in blood vessels, promoting inflammation and endothelial dysfunction. Cytokine release triggers an inflammatory and immune response, where MMPs are involved. (**B**) MMP in heart failure and post-MI remodeling. MMPs play a dual role by aiding tissue remodeling and repair post-MI but also contributing to adverse ventricular remodeling, which can lead to heart failure. Intracellular target structures for MMP-2 and MMP-9 are depicted. MMP-2 and MMP-9 interact with sarcomeric proteins (troponin I, titin, MLC-1) disrupting contractile function. MMP-2 and MMP-9 also cause mitochondrial impairment, affecting energy production and impairing cardiac metabolism. Extracellular matrix (ECM); myosin light chain (MLC-1); sarcoplasmic/endoplasmic reticulum Ca^2+^-ATPase 2a (SERCA2a); junctophilin-2 (JPH-2).

MMPs play a crucial role in vascular inflammation and the remodeling of atherosclerotic plaques, contributing to plaque rupture by degrading the fibrous cap. Damage to endothelial cells (ECs) caused by pathogens or oxidative stress triggers an inflammatory response, encouraging leukocyte adhesion and migration into the vessel wall [1]. This activates MMPs, leading to vascular smooth muscle cell (VSMC) proliferation, the disruption of tight junction proteins, and the degradation of elastin and collagen, resulting in overall vascular dysfunction [1] (Figure 2). Thus, MMPs influence all phases of atherosclerosis. The balance between MMPs and their endogenous inhibitors, TIMPs, regulates tissue remodeling, repair, and resorption [36,48]. Disruptions in this balance, often driven by oxidative stress and inflammation, can lead to excessive MMP activity, promoting the progression and instability of atherosclerotic plaques [1]. Increased MMP activity, especially in the vulnerable regions of plaques, contributes to plaque growth and destabilization [46,49]. This, combined with macrophage foam cell apoptosis and fibrous cap degradation, increases the likelihood of plaque rupture, which can result in the formation of an occlusive thrombus and potentially trigger an MI [48].

Interstitial collagenase activity, particularly targeting collagens type I and III, which are resistant to most proteinases, is primarily attributed to MMP-1 [37]. Studies show that MMP-1 is produced by macrophages in atherosclerotic plaques, where it colocalizes with degraded collagen in vulnerable areas prone to rupture [37,50]. Macrophages and smooth muscle cells express MMP-1 in response to proatherogenic factors like shear stress, oxidized low-density lipoprotein (LDL) cholesterol, and inflammatory cytokines [51]. Additionally, platelets release MMP-1 when exposed to thrombin, allowing MMP-1 to activate platelets independently through the protease-activated receptor 1 (PAR1) pathway, bypassing the usual thrombin-mediated process [50]. These mechanisms suggest that MMP-1 contributes to atherosclerosis by degrading the vessel wall matrix and promoting platelet activation, which may lead to plaque destabilization and thrombotic complications [50]. Elevated levels of MMP-2, unique among MMPs due to its collagen-binding domain, are found in atherosclerotic plaques, and recent studies have shown that extracts from human carotid plaques, rich in active MMP-2, can induce platelet aggregation, an effect preventable by MMP-2 inhibitors [8,52]. Increased MMP-2 activity in plaques is linked to a higher risk of ischemic cerebrovascular events, highlighting its key role in platelet thrombus formation and plaque instability [52].

MMP-3 (stromelysin-1) also plays a critical role in the development of atherosclerotic plaques by contributing to ECM turnover in the vascular wall, which can lead to plaque rupture [36]. It has been suggested that MMP-3 levels vary according to the severity of CAD, as it is involved in plaque destabilization and vascular remodeling through the breakdown of collagen in the fibrous cap [36]. MMP-3 also regulates VSMCs’ migration by activating MMP-9, and its expression may precede that of MMP-9 [36].

Produced by activated macrophages, MMP-7, or matrilysin, efficiently degrades various matrix components like proteoglycans, elastin, and fibronectin, being associated with the apoptosis of VSMCs and foam cells in the necrotic core of plaques, playing a significant role in plaque destabilization [53]. It also cleaves N-cadherin, a protein responsible for cell–cell junctions in VSMCs, promoting their apoptosis and contributing to plaque rupture [54]. Immunohistochemical studies have shown that MMP-7 is primarily located in macrophages within areas of disorganized collagen, indicating its influence on collagen plaque structure [39]. Elevated MMP-7 levels in CAD suggest its potential as a biomarker for the disease [55].

Another key enzyme involved in ECM remodeling is MMP-9 as it degrades denatured collagens [8]. Histopathological studies reveal that MMP-9 is primarily found in the necrotic core and fibrous cap of atherosclerotic plaques, with increased levels and activity in unstable plaques compared to stable ones [47]. Inflammatory cells, including monocytes, macrophages, neutrophils, and foam cells, secrete MMP-9, particularly under pathological conditions when proinflammatory factors are present [47]. An imbalance between MMP-9 and its inhibitor, TIMP-1, as well as genetic polymorphisms of MMP-9, contributes to plaque instability and CAD [38]. A meta-analysis investigating low-grade inflammation in CAD patients found increased levels of MMP-9 [56]. Elevated serum MMP-9 levels were associated with greater intimal thickness and plaque instability [1]. Patients with CAD, angina, or MI also exhibited higher plasma levels of MMP-9 and its inhibitor TIMP-1 [36]. Notably, MMP-9 levels were particularly high in patients with unstable unruptured plaques and ruptured plaques compared to stable ones, indicating its role in plaque stability [36]. Therefore, MMP-9 is also suggested as a potential biomarker for assessing atherosclerotic plaque stability and predicting future cardiovascular and cerebrovascular events [47], serving as an early indicator of disease severity and a potential risk factor for complications in CAD patients [36].

MMP-12, a metalloelastase initially identified in the alveolar macrophages of smokers, has been shown to negatively impact plaque stability in a mouse model of brachiocephalic artery atherosclerosis [57]. The use of an MMP-12 inhibitor resulted in a significant reduction in plaque formation, necrosis, calcification, and macrophage apoptosis, leading to thinner fibrous plaques and the slower progression of atherosclerosis [1]. MMP-13, a collagenolytic metalloproteinase that is absent in platelets but upregulated in atherosclerotic and inflammatory tissues, has been found to reduce thrombus formation on fibrillar collagen under flow conditions by partially digesting collagen molecules (monomeric collagen chains), thereby disrupting platelet–collagen interactions [52]. This strongly suggests that MMP-13 may inhibit platelet recruitment at ruptured plaques [52].

### 3.2. MMPs in Myocardial Infarction

Myocardial infarction is among the leading causes of cardiovascular-related deaths [19,21]. A precursor to MI is the formation of an unstable plaque, known as a trombus, whose properties are closely linked to the ECM [11,21]. The ECM’s composition, architecture, and functions are crucially dependent on its remodeling capacity and ability to maintain structural integrity [11,21]. The remodeling properties of MMPs are also important in the progression to HF following MI [40]. After an MI, the heart undergoes a process of left ventricular remodeling, characterized by changes in its size, shape, and function [40]. While this remodeling process is beneficial in the early stages, by enabling repair, excessive or uncontrolled remodeling can lead to pathological consequences such as ventricular dilation and HF [21,40], the most important MMPs in this circumstances being MMP-2 and MMP-9 [12,58] (Figure 2).

The first member of the MMPs family to be isolated from myocardial cells was MMP-2, which is physiologically present under normal conditions. However, its concentration increases significantly during ischemic processes, rising above baseline levels [14]. Furthermore, elevated levels are also associated with other cardiovascular conditions, including HF and left ventricular dysfunction [40]. The pathophysiology of myocardial IR injury involves a complex interplay of microcellular processes, such as inadequate excitation–contraction coupling, calcium overload, and heightened oxidative stress, all of which are influenced by ECM remodeling [19]. MMP-2 undergoes both activation and inhibition, depending on its localization and the stage of the IR injury [19]. During the ischemic phase, there are elevated levels of peroxynitrite activate MMP-2, contributing to tissue damage [19]. In contrast, inhibiting MMP-2 activity has been shown to enhance cardiac function, improve cardiomyocytes reperfusion, and prevent junctophilin-2 (JPH-2) proteolysis, a membrane protein critical for calcium release in cardiomyocytes [19].

Elevated MMP-9 levels in the bloodstream have also been associated with poor prognosis in patients with MI and unregulated activity, resulting in pathological changes, including ventricular dilation and progression to HF [40]. Consequently, these levels serve as valuable biomarkers for predicting adverse outcomes, including HF and post-infarction complications [40]. Another metalloproteinase expressed by macrophages within infarcted heart tissue is MMP-7, which plays a significant role in mediating the inflammatory response and also facilitating repair processes by the breakdown of various ECM components [39,40].

Consequently, a comprehensive study conducted by Kaminski et al. analyzed MMP expression in mice following MI [30]. Their findings revealed that the expression of the messenger ribonucleic acid (mRNA) of nearly all MMPs increased in the hearts of mice, except for 6 specific MMPs (MMP-15, -17, -19, -21, -23b, and -26) out of the total 23 MMPs [30]. Targeting MMP activity presents a promising therapeutic approach, though further research is required to develop safe and effective strategies to modulate these enzymes in the context of MI [7].

### 3.3. MMPs in Heart Failure

The pivotal role played by metalloproteinases in the pathophysiology of HF consists primarily of regulating ECM remodeling, contributing to both myocardial injury and repair [11,40,41]. The ECM provides essential structural support to cardiac cells, and its remodeling is critical for preserving normal heart functions [7,59]. However, excessive or inappropriate ECM degradation, which can occur in HF, contributes to left ventricular dysfunction and pulmonary hypertension (PH) progression [11,21,40]. The pathological activation of MMPs in HF accelerates the breakdown of ECM components, compromising the structural integrity of the cardiac tissue [60].

The key MMPs implicated in HF include MMP-1, MMP-2, MMP-3, MMP-9, and MMP-14 [40]. Furthermore, in HF, their expression and activity become dysregulated, driving pathological changes in the cardiac tissue structure and function [40]. Chronic HF, particularly following MI, initiates a process in which MMPs play a significant role in disrupting normal myocardial function and promoting the development of fibrotic scar tissue [21,40]. After MI, elevated MMP-1 activity contributes to the degradation of collagen in the infarcted tissue, resulting in fibrosis and adverse cardiac remodeling that compromise myocardial structural integrity and impair contractile function [21]. The interaction between MMPs and the Renin–Angiotensin–Aldosterone System (RAAS) is another key aspect of this topic, as RAAS inhibitors play a critical role in the pathogenesis of HF by mitigating inflammation, fibrosis, and remodeling [41,61]. In HF, due to inflammation and oxidative stress, MMP-2 is overactivated, resulting in excessive ECM breakdown, myocardial dilation, and fibrosis, all of which contribute to impaired ventricular function [41]. At a molecular level, the reduced cardiac contractility is explained by degrading components of the contractile apparatus, such as troponin I and light chain myosin 1 [18,41] (Figure 2). Given that elevated plasma levels of MMP-2 have been observed in patients with congestive HF, regardless of the underlying cause (such as acute MI, dilated cardiomyopathy, or valvular disease), MMP-2 may serve as a potential biomarker [41]. Higher MMP-2 levels are also associated with a worse prognosis in HF patients, particularly those in NYHA classes II–IV [41].

Regarding HF, elevated MMP-9 levels are linked to increased myocardial damage, inflammation, and the progression of diastolic dysfunction. Consequently, it is recognized as a major biomarker for cardiac muscle degradation, monitoring disease progression and therapeutic efficacy [62,63]. In HF’s pathophysiologic milieu, its expression is upregulated in response to cytokines (e.g., TNF-α, IL-1β), hypoxia, ROS, growth factors, and is involved in the degradation of ECM components, as well as the recruitment of inflammatory cells to the site of injury [62].

In summary, MMPs play a pivotal role in the ECM remodeling process in HF, as their dysregulated activity contributes to the structural and functional alterations characteristic of the condition, including myocardial fibrosis, ventricular dilatation, impaired contractility, and relaxation [64,65]. While MMPs are essential for normal tissue repair, their excessive or uncontrolled activation during HF exacerbates disease progression [64,65]. Modulating MMP activity presents a promising therapeutic approach; however, further research is required to develop effective and selective strategies for regulating MMP activity in the context of HF [64,65].

### 3.4. MMPs in Valvular Heart Disease: Focus on Aortic Stenosis

MMPs that play a role in the progression of aortic stenosis can be categorized into three distinct functional groups: stromelysins, collagenases, and gelatinases. These enzymes are indicative of pronounced ECM remodeling occurring within the aortic valve [66]. Aortic stenosis is a pathophysiological condition defined by the restricted opening of the valve leaflets, which leads to increased afterload on the left ventricle, subsequent compensatory hypertrophy of the left ventricle, and compromised hemodynamic flow from the left ventricle to systemic circulation [66]. The primary lesions associated with aortic stenosis include significant changes in the ECM (notably fibrosis), along with inflammation and calcification of the valve leaflets, all of which markedly contribute to the stiffening and dysfunction of the valve [66]. Calcific aortic valve disease (CAVD), the primary cause of aortic stenosis, is defined by several pathological processes, including lipid deposition, inflammation, calcification, neoangiogenesis, and compromised endothelial function, factors that are frequently associated with atherosclerosis [42,67]. It has been confirmed that several MMPs are engaged in aortic valve tissue remodeling, namely MMP-12, MMP-9, and MMP-1, which exhibit significant upregulation in cases of calcific aortic valve stenosis [43].

The elevated expression of MMP-1, a collagenase that possesses the ability to cleave the intact triple-helix of fibrillar collagen, has been observed in the early stages of aortic stenosis [68]. In individuals with mild aortic stenosis, there was a significant relationship observed between the left ventricular end-diastolic volume index and MMP-1 levels, which were also linked to left ventricular volume overload and impaired diastolic function [69,70]. Conversely, patients suffering from severe aortic valve stenosis exhibited the lowest concentrations of MMP-1, possibly reflecting a shift from active remodeling to disease progression, leading towards valvular calcification [71]. Similarly, MMP-9, which displays a strong affinity for denatured collagen fibers and proteins found in basement membrane proteins, also appears to be downregulated in advanced disease stages [72]. This reduction, alongside elevated levels of TIMP-1, thus suggests a stable remodeling state characterized by a predominance of pro-fibrotic cardiac ECM accumulation surpassing turnover and degradation [72].

MMPs are also implicated in the osteogenic processes driving valve calcification. MMP-12, primarily recognized for its capacity to degrade elastin, an essential protein within the extracellular matrix that is abundantly found in arterial walls, is particularly prevalent in the calcified and stenotic regions of the aortic valve [43]. Initially produced as an inactive precursor, MMP-12 transitions to its active form through the autolytic cleavage of its NH2-terminal domain [43]. Once activated, MMP-12 can stimulate the synthesis of several pro-osteogenic factors in aortic valve interstitial cells, including runt-related transcription factor 2 (Runx2) and bone morphogenetic protein 2 (BMP-2), a significant osteogenic factor identified in calcified tissue along with alkaline phosphatase (ALP), thus facilitating mineralization and the formation of bone-like lesions within the valve [43]. Runx2, an essential transcription factor involved in the osteogenic transdifferentiation process of VSMCs, is generally absent from healthy aortic valves [73,74]. Additionally, MMP-10 is upregulated in calcified valves and co-expressed alongside calcification markers such as Runx2 and sex-determining region Y (SRY)-box 9 (Sox-9) within stenotic valves [42]. Its activation under inflammatory stimuli contributes to osteogenic signaling and the release of proinflammatory mediators such as tumor necrosis factor (TNF) α, IL-6, and IL-1β, creating a self-perpetuating inflammatory loop that contributes to progressive calcification [42].

Surprisingly, recent studies have highlighted sexual dimorphism in MMP expression in patients with aortic stenosis, emphasizing the differences in histological and molecular patterns [75]. Despite similar hemodynamic severity, valves from female patients have demonstrated reduced levels of inflammation, oxidative stress, apoptosis, and calcification when compared to their male counterparts [75]. However, females exhibit abnormal ECM remodeling, driven by the increased activity of MMP-1 and MMP-9 and decreased TIMP-2 levels, possibly amplifying MMP-2 activity [75]. This imbalance may result in the excessive degradation of collagen type I and altered valve matrix composition [75].

Together, these findings underscore the multifaceted roles played by MMPs in the pathophysiology of aortic valve disease, mediating ECM remodeling, driving calcification, and contributing to inflammatory and sex-specific mechanisms.

## 4. Potential Therapeutic Targets

Given their critical involvement in cardiovascular pathology, MMPs have emerged as potential therapeutic targets for limiting disease progression and mitigating adverse cardiovascular remodeling. Strategies aimed at modulating MMP activity range from direct pharmacological inhibition to the regulation of endogenous MMP modulators such as TIMPs. Additionally, gene therapy and anti-inflammatory approaches are being explored to achieve more targeted and effective interventions. This section provides an overview of the current and emerging therapeutic strategies designed to regulate MMP activity and improve cardiovascular outcomes.

### 4.1. MMP Inhibitors

Suppressing MMP activity is considered a possible strategy for preventing atherosclerotic plaque destabilization, as this inhibition may help reduce excessive collagen breakdown and preserve the structural integrity of the fibrous cap [76]. First-generation MMP inhibitors contain a hydroxamate zinc-binding group, which exhibits a strong affinity for the Zn^2+^ ion within the catalytic site of MMPs [77]. Preliminary research studies involving zinc-chelating inhibitors, such as Batimastat and Marimastat, sought to reduce MMP activity in different tissues, but these trials encountered significant setbacks due to unintended effects, such as musculoskeletal complications and impaired tissue regeneration [76,77]. Consequently, efforts have shifted toward developing more selective inhibitors targeting specific MMPs associated with plaque rupture while avoiding interference with other MMPs critical for tissue stability [76]. Notably, the systemic administration of Batimastat, also known as BB-94, decreased aortic dilatation and medial elastin degradation in rat models of elastase-mediated abdominal aortic aneurysms [78]. Preclinical studies suggest that targeting MMP-2 inhibition could be a promising therapeutic strategy for HF [79]. ONO-4817, a selective MMP-2 inhibitor, improved contractile dysfunction, which correlates with reduced MMP-2 activity and titin proteolysis in a model of IR injury [79]. Additionally, it has shown potential in mitigating left ventricular remodeling and myocardial fibrosis in mice treated with HF induced after doxorubicin administration [80]. These findings support the therapeutic potential of MMP-2 inhibition through ONO-4817, as it effectively reduces lesion severity and disease progression while avoiding the inhibition of MMP-1, associated with adverse effects linked to hydroxamate-based inhibitors [80]. Another study where treatment with ONO-4817 significantly inhibited MMP-9 activity demonstrated attenuated left ventricular remodeling within one day after myocardial infarction, highlighting its potential therapeutic role in post-MI complications [47]. Therefore, while ONO-4817 shows the preferential inhibition of MMP-2, its potential off-target effects on MMP-9 remain relevant and should be considered in experimental interpretations.

The promise of MMP-2 inhibition leads to developing selective MMP-2 inhibitors, MMPI-1154 and MMPI-1260, that demonstrated efficacy in reducing infarct size by decreasing MMP-2 activity [81,82]. However, despite their potential, these inhibitors have not been tested in other preclinical HF models, nor have they been evaluated in clinical studies [81,82]. Additionally, an in vivo acute infarction model explored the encapsulation of MMP-2 siRNA in a hydrogel to enhance cellular uptake. This approach yielded positive effects on cardiac hemodynamics, as MMP-2 reduction helped preserve ejection fraction [83].

Morin, a naturally sourced polyphenol with antioxidant properties, was found to inhibit MMP-9 activity in vascular smooth muscle cells, thereby suppressing endothelial-to-mesenchymal transition that leads to the formation of compromised vascular endothelial cells [84,85]. Among natural MMP inhibitors, TIMP-3 stands out due to its exclusive high affinity for proteoglycans in the extracellular matrix [59]. TIMP-3 is believed to function as an endogenous anti-inflammatory agent, mainly through the inhibition of ADAM17, thus preventing the ADAM17-mediated release of soluble TNF-α, a key inflammatory cytokine [59]. The therapeutic potential of delivering recombinant TIMP-3 (rTIMP-3) using a hydrogel system in a porcine model of MI was studied by Eckhouse et al. The sustained release of rTIMP-3 within the infarcted myocardial region significantly reduced adverse left ventricular remodeling and interstitial MMP activity. Additionally, this targeted therapy led to a marked reduction in proinflammatory cytokines and the increased presence of smooth muscle actin, indicative of enhanced myofibroblast proliferation [86].

Doxycycline, a broad-spectrum antibiotic and tetracycline derivative, exerts anti-inflammatory effects by inhibiting MMP-9 and MMP-2 activity within the aortic aneurysm wall [78,87]. This results in reduced neutrophil infiltration, thereby lowering inflammation and halting the progression of abdominal aortic aneurysms [87]. Additionally, Doxycycline has been proven to substantially mitigate left ventricular remodeling in ST-elevation myocardial infarction (STEMI) patients and improve ejection fraction [80,88]. In another study, Doxycycline was proposed as a potential therapeutic approach to counteract hypertension-induced maladaptive cardiac remodeling and dysfunction, thus potentially delaying the onset of HF [89]. Notably, it has been demonstrated to prevent the transition from concentric to eccentric left ventricular hypertrophy in hypertension, even without a reduction in systolic blood pressure [41,89]. This effect was correlated with decreased MMP-2 activity and the diminished degradation of troponin I and dystrophin, thereby preserving the structural integrity of cardiomyocytes and enhancing contractility [41,89].

Carvedilol, a non-selective β-adrenergic antagonist with α-blocking and antioxidant properties, is widely prescribed for managing HF, hypertension, and left ventricular dysfunction post-MI. In an experimental autoimmune myocarditis rat model, this drug was found to suppress MMP-2 activity, leading to a decrease in troponin I breakdown, myofilament degradation, and cardiac structural damage [90]. Similarly, Verapamil, another drug indicated for hypertension treatment, has been found to inhibit MMP-2 activity by mitigating oxidative stress [41]. As a first-generation calcium channel blocker, Verapamil also helps decrease left ventricular concentric hypertrophy by the downregulation of calpain-1, a calcium-dependent cysteine protease which plays a key role in the development of cardiac hypertrophy and fibrosis through the activation of nuclear factor kappa B (NFκB) and transforming growth factor-β (TGF-β) [91].

Statins are a class of drugs primarily used to lower cholesterol levels by inhibiting the enzyme 3-hydroxy-3-methylglutaryl-coenzyme A (HMG-CoA), a key factor in the mevalonate metabolic cascade responsible for cholesterol synthesis in the liver [92]. Beyond their lipid-lowering effects, statins also exhibit pleiotropic effects, including the modulation of signaling pathways that result in the inhibition of MMP activity, decreasing vascular inflammation [92]. It has been reported that Atorvastatin improved fibrous cap thickness and plaque stability while reducing MMP-9 levels among individuals with CAD [92,93]. Furthermore, by normalizing endothelial lysyl oxidase (LOX) enzyme expression and inhibiting the secretion of MMPs from inflammatory cells, these lipid-regulating drugs have demonstrated their role in enhancing plaque collagen levels [94]. In patients with HF with reduced ejection fraction (HFrEF) following an acute MI, clinical studies revealed that treatment with statins, such as Atorvastatin, Rosuvastatin, and Pravastatin, led to reduced serum MMP-2 levels, which were accompanied by lower mortality rates and fewer hospital readmissions [41]. The activation of MMP-2 and MMP-9 has been proven to be inhibited by Simvastatin, along with Lovastatin and Atorvastatin, through the downregulation of myosin phosphatase target subunit 1 (MYPT1) and myosin light chain (MLC) phosphorylation [95].

In a rat model of MI, Lisinopril, a representant of angiotensin-converting enzyme inhibitors (ACEi), demonstrated significant suppression activity on MMP-9 [96]. Moreover, in patients with HF secondary to hypertensive, it has been shown that the combination of Sacubitril and Valsartan could enhance vascular endothelial function and decrease MMP-9 levels [97]. In the process of aging, angiotensin II (Ang II) and ACE levels increase in the myocardium, where Ang II directly contributes to myocyte hypertrophy, left ventricular stiffness, apoptosis, fibrosis, and reduced diastolic performance [98]. MMP-9 is a key contributor to the age-related increase in left ventricular stiffness, playing a crucial role in preserving vessel integrity as well [98]. ACEi is able to reduce MMP-9 activity through a direct interaction with its catalytic domain, which partly accounts for the positive effects on cardiac aging [98,99].

### 4.2. Gene Therapy

MicroRNAs (miRNAs), also classified as non-coding RNAs, are usually transcribed from deoxyribonucleic acid (DNA) as primary miRNAs, which are later processed into precursor miRNAs and ultimately mature miRNAs [100]. Modulating miRNA expression holds promise as a therapeutic approach for patients with aortic stenosis and as potential strategies for miRNAs’ overexpression, including miRNA mimics, miRNA expression vectors, and small molecules [100]. For example, miR-451a mimics, which are synthetic RNA molecules that replicate the function of endogenous miR-451a, have been demonstrated to counteract the increased expression of macrophage migration inhibitory factor (MIF), a key regulator of cardiac inflammatory pathways [101]. Consequently, MMP-2 and MMP-9 levels were reduced in abnormal cardiomyocytes in response to pathological stress [101].

Adeno-associated virus (AAV) has emerged as a promising vector for gene therapy due to its non-pathogenic nature, basic structure, genetic composition, and lower immunogenicity in comparison to other viruses [102]. The AAV9-based provector is initially in an inoperative state, and it can be activated by MMP-2 and MMP-9 [102]. In an in vivo model of MI, this provector demonstrated the ability to deliver transgenes specifically to regions of the damaged heart exhibiting high MMP activity [102].

In summary, while MMPs hold promise as therapeutic targets for CVD, further research and large-scale clinical trials are necessary to validate their inhibitors. Additionally, developing highly selective inhibitors can enhance efficacy while minimizing off-target effects (Table 3).

To ensure successful clinical trials, key challenges must be addressed, including selecting the right patient population, defining appropriate trial endpoints, optimizing dosage and treatment duration, ensuring monitoring, and enrolling a sufficient number of participants for statistical reliability. A deeper understanding of MMPs’ intra- and extracellular functions is also essential to guide the development of targeted therapies. While MMP inhibitors offer significant therapeutic potential, optimizing their selectivity and conducting well-structured clinical trials will be key to unlocking their full benefits for CVD treatment (Table 4).

## 5. Conclusions

MMPs are important modulators of ECM remodeling and have been implicated in various CVDs. Beyond ECM degradation, MMPs are involved in inflammatory pathways, vascular remodeling, lipoprotein metabolism, and intracellular signaling processes [1,2]. However, their functions are highly context-dependent, with varying roles according to disease stage and tissue environment. While dysregulated MMP activity is associated with pathological changes in conditions such as atherosclerosis, myocardial infarction, heart failure, and aortic stenosis, these relationships are often correlative, and direct causal links remain incompletely understood [3,4,30]. An important consideration in interpreting the findings from MMP knockout models is the extensive substrate overlap among MMP family members, which introduces a significant limitation that is often underacknowledged. Many MMPs family members share the ability to degrade similar components of ECM. As a result, the deletion of a single MMP gene may not fully disrupt proteolytic action as a compensatory mechanism. This poses a challenge in attributing observed phenotypic outcomes solely to the absence of a particular MMP, especially in complex diseases such as atherosclerosis and post-MI remodeling.

Despite extensive research, translating MMP-targeting strategies into effective clinical therapies remains a challenge. First-generation broad-spectrum MMP inhibitors have yielded disappointing results due to off-target effects, prompting interest in more selective approaches including TIMPs, gene-based interventions, microRNA regulation, and nanotechnology-based drug delivery [76,77,105,106]. These newer strategies may offer greater specificity and reduced toxicity, though they remain in the early stages of development.

Future research should focus on unraveling the intricate regulatory mechanisms governing MMP activity, identifying patient-specific MMP expression profiles, and developing personalized therapeutic strategies. Advanced technologies, including proteomics and single-cell RNA sequencing, may provide deeper insights into MMP function in different cardiovascular conditions [105,106].

In conclusion, while MMPs meaningfully contribute to cardiovascular pathology, their effects are not always causative. Continued efforts to refine MMP-targeted therapies, integrate them into personalized medicine, and explore their broader systemic effects will be essential for improving clinical outcomes in patients with cardiovascular diseases.

## Figures and Tables

**Table 1:** Classification of MMPs and their substrates.

Refs.	MMP Family	MMP Members	Main Substrates	Biological Function
[4,7,9]	Collagenases	MMP-1, MMP-8, MMP-13, MMP-18	Fibrillar collagens (I, II, III)	Collagen breakdown in tissue remodeling
[4,7,9]	Gelatinases	MMP-2, MMP-9	Denatured collagens, gelatin	Degradation of basement membrane
[4,7,9]	Stromelysins	MMP-3, MMP-10, MMP-11	ECM proteins, proteoglycans	Tissue remodeling, inflammation
[4,7,9]	Matrilysins	MMP-7, MMP-26	Laminin, elastin, fibronectin	Wound healing
[4,7,9]	MT-MMPs	MMP-14, MMP-15, MMP-16, MMP-17	ECM components, other MMPs	Cell invasion, pericellular proteolysis
[4,7,9]	Other MMPs	MMP-19, MMP-20	Elastin, dentin matrix proteins	Vascular remodeling

**Table 2:** Role of MMPs in cardiovascular diseases.

Refs.	Cardiovascular Disease	MMPs Involved	Target
[8,36,37,38]	Atherosclerosis	MMP-1, MMP-2, MMP-3, MMP-9	Plaque degradation, vascular inflammation
[19,21,39]	Myocardial Infarction	MMP-2, MMP-7, MMP-9	ECM breakdown
[30,40,41]	Heart Failure	MMP-2, MMP-9, MMP-14	Myocardial remodeling, fibrosis
[42,43]	Aortic Stenosis	MMP-1, MMP-9, MMP-12	Valve calcification, ECM degradation

**Table 3:** MMP inhibitors and their mechanisms of action.

MMP Inhibitor Type	Examples	Mechanism of Action	Limitations
Synthetic inhibitors [76,77,78]	Batimastat, Marimastat	Zinc-chelation, broad-spectrum inhibition	Toxicity, off-target effects
Synthetic inhibitors [79,80]	ONO-4817	Selective MMP-2 inhibitor	Limited impact on other MMPs
Synthetic inhibitors [81,82]	MMPI-1154 and MMPI-1260	Selective MMP-2 inhibitor	
Non-selective β-adrenergic antagonist [90]	Carvedilol	Suppress MMP-2 activity	
Calcium channel blocker [41]	Verapamil	Inhibit MMP-2 activity	
Tetracyclines [78,87]	Doxycycline	Downregulation of MMP expression	Limited specificity
Statins [41,92,93,95]	Atorvastatin	Reducing MMP-9 levels	
	Atorvastatin, Rosuvastatin, and Pravastatin	Reduced serum MMP-2 levels	Limited specificity, potential drug interactions, short duration
	Atorvastatin, Simvastatin, and Lovastatin	Inhibit activation of MMP-2 and MMP-9	
ACEi [96,98]	Lisinopril	Suppression activity on MMP-9	Dose-dependent effects
Natural compounds [84,85]	Morin	Antioxidant-mediated MMP suppression	Low bioavailability
Gene therapy [102]	AAV9-based vectors, miRNA-451a	Inhibits MMP-2/MMP-9 expression	Indirect MMP inhibition, stability, and uptake variability

**Table 4:** Summary of main MMP studies in CVDs.

Ref.	Cardiovascular Disease	Subjects	Notable Effects
[44]	Atherosclerosis and coronary artery disease	Human Peripheral Blood Monocytes (PBMCs)	TIMP-1–CD74 axis in inflammatory/atherogenic responses
[45]		ApoE^−/−^ mice deficient in human MMP1 ortholog, MMP1a.	Role for MMP1a in atherosclerotic lesion development
[46]		Age- and gender-matched case–control study	MMP-9 and the MMP-9/TIMP-1 molar ratio may be valuable in acute coronary syndrome diagnosis and prognosis
[36]		472 patients with CAD	Combination of MMP-9, TIMP-2, and Apo-CIII values (‘CAD aggravation panel’) characterizes theseverity of CAD
[48]		ApoE-deficient mice with concomitant deletion of MMP-7, MMP-9, MMP-12, or TIMP-1	MMP-7 deficiency increased incidence of sudden deathMMP-12 deficiency promoted survivalMMP-9 or TIMP-1 deficiency had no effect
[49]		Serum protein levels of MMP-1, MMP-3, and MMP-12 from patients with carotid atherosclerosis (CAS)	MMP-1, MMP-3, and MMP-12 were significantly increased and had significantly positive correlations with the occurrence of CAS
[37]		Serum concentration of MMP 1 from 300 CAD patients	MMP 1 serum levels and polymorphism as potential prognostic markers for future cardiovascular events
[50]		364 male patients	Elevated levels of MMP-1 are associated with an increased risk of long-term all-causemortality
[8]		MMP-9 transgenic (Tg) rabbits	Macrophage-derived MMP-9 facilitates the infiltration of monocyte/macrophages, enhancing the progression of atherosclerosis
[39]		Blood samples from 32 subjects with stable coronary heart disease (CHD) and elevated lipoprotein(a) (Lp(a)	MMP-9 is a strong independent predictor ofatherosclerotic plaque instability in stable CHD patients
[38]		132 patients who underwent coronaryangiography	High levels of TNF-α and IL-6 could influence the MMP-9/TIMP-1 balance and lipid metabolism, leading to plaque formation/rupture
[19]	Myocardial Infarction	Male Sprague–Dawley rats	Degradation of JPH-2 by MMP-2 is an early consequence of myocardial IR injury
[103]		50 patients	Pro-MMP-9 activity reduced by 50% after Doxycycline 20 mg twice daily
[14]	IR injury	Conditioned media from human fibrosarcoma HT1080 cellSprague–Dawley neonatal ratsventricular cardiomyocytes (NRVMs)	Nuclear MMP-2 activity indicates lamin proteolysis
[11]	Heart failure	Humans	MMP-2 may reflect aberrant ECMremodeling involved in the pathophysiology of HF and associated pulmonary hypertension
[60]		Venous blood samples from patients with HF	Potential inhibitory effect ofantihypertensive treatment on pro-MMP-2 activity
[18]		Venous blood sample from 101 patients with chronic HF	Elevated levels of MMP-2 and TIMP-2were found in serum from patients with chronic kidney diseaseSerum levels of MMP-2 were correlated with the degree of kidney failure
[62]		Male Sprague Dawley rats	Beneficial outcome of MMP-9 inhibition on pathological cardiac remodeling
[104]		4693 participants from atherosclerosis risk in community study	Higher MMP-2 levels were associated with HF and diastolic dysfunction
[42]	Valvular heart disease	Blood samples from patients undergoing valvereplacement	MMP-10 plays a central role in calcificationin AS through Akt phosphorylation
[67]		ApoE^−/−^ mice fed a Western diet (WD)	MMP-targeted imaging detects valvular inflammation and remodeling in CAVD
[43]		Human aortic valve interstitial cells (AVICs)	MMP-12 induces pro-osteogenic responses in AVICs by activation of p38 MAPK signaling pathway
[71]		Venous blood samples from patients with aortic stenosis	MMP-1 levels could indicate the development ofcalcinosis in severe stenosis
[72]		Patients with aortic stenosis	MMP-9 alterations reflect the switch of extra-valvular cardiac damage from left ventricular to left atrial involvement
[75]		238 patients with severe aortic stenosis undergoing surgical valve replacement	Women exhibited increased MMP-1 and decreased TIMP-2 expression

## Data Availability

No new data were created or analyzed in this study.
